# DEK over expression as an independent biomarker for poor prognosis in colorectal cancer

**DOI:** 10.1186/1471-2407-13-366

**Published:** 2013-07-31

**Authors:** Lijuan Lin, Junjie Piao, Wenbin Gao, Yingshi Piao, Guang Jin, Yue Ma, Jinzi Li, Zhenhua Lin

**Affiliations:** 1 Department of Pathology, Yanbian University College of Medicine, Yanji 133002, China; 2 Department of Medical Imaging, Eastern Liaoning University College of Medicine, Dandong 118002, China; 3 Department of Oncology, Affiliated Zhongshan Hospital of Dalian University, Dalian 116000, China; 4 Cancer Research Center, Yanbian University, Yanji 133002, China; 5 Department of Internal Medicine, Yanbian University Affiliated Hospital, Yanji 133000, China

**Keywords:** Colorectal cancer, DEK, Immunohistochemistry, Survival analysis

## Abstract

**Background:**

The DEK protein is related to chromatin reconstruction and gene transcription, and plays an important role in cell apoptosis. High expression levels of the human DEK gene have been correlated with numerous human malignancies. This study explores the roles of DEK in tumor progression and as a prognostic determinant of colorectal cancer.

**Methods:**

Colorectal cancer specimens from 109 patients with strict follow-up, and colorectal adenomas from 52 patients were selected for analysis of DEK protein by immunohistochemistry. The correlations between DEK over expression and the clinicopathological features of colorectal cancers were evaluated by Chi-square test and Fisher’s exact tests. The survival rates were calculated by the Kaplan-Meier method, and the relationship between prognostic factors and patient survival was also analyzed by the Cox proportional hazard models.

**Results:**

DEK protein showed a nuclear immunohistochemical staining pattern in colorectal cancers. The strongly positive rate of DEK protein was 48.62% (53/109) in colorectal cancers, which was significantly higher than that in either adjacent normal colon mucosa (9.17%, 10/109) or colorectal adenomas (13.46%, 7/52). DEK over expression in colorectal cancers was positively correlated with tumor size, grade, lymph node metastasis, serosal invasion, late stage, and disease-free survival- and 5-year survival rates. Further analysis showed that patients with late stage colorectal cancer and high DEK expression had worse survival rates than those with low DEK expression. Moreover, multivariate analysis showed high DEK expression, serosal invasion, and late stage are significant independent risk factors for mortality in colorectal cancer.

**Conclusions:**

DEK plays an important role in the progression of colorectal cancers and it is an independent poor prognostic factor of colorectal cancers.

## Background

The DEK gene, on chromosome 6, encodes a 375-amino acid protein with an estimated molecular weight of 43kD. It has not been classified into any known protein family [1-3]. Human DEK is an abundant nuclear protein with important functions in the architectural regulation of chromatin assembly. It was originally identified as a fusion with CAN/NUP214 nucleoporin in a subset of acute myeloid leukemia (AML) patients, and was named on the basis of the initials of the patient DK [4,5].

Since its discovery as the target of the t(6;9) translocation in a subset of AML patients, DEK has been repeatedly associated with tumor development. High expression levels of the human DEK gene have been correlated with numerous human malignancies such as glioblastoma, melanoma, breast cancer, ovarian cancer and hepatocellular carcinoma [1,4,6,7]. To date, no mutations have been reported in the coding sequence of human DEK. However, various other regulatory mechanisms have been identified at the DNA, RNA, and protein levels [6-8]. Intracellularly, DEK has been described to induce DNA supercoiling, DNA replication, RNA splicing and transcription in vitro [4,8,9]. Wise-Draper et al. demonstrated that DEK suppresses cellular senescence, apoptosis and differentiation, and promotes epithelial transformation in vitro and in vivo [10]. Datta et al. recently reported that oncoprotein DEK is upregulated in bladder cancer tissues in comparison with normal counterparts as determined by western blot. Indeed, DEK protein was shown to be present in the voided urine of patients with both low- and high-grade bladder cancer, suggesting that DEK could be used as a biomarker for detection of this cancer using patient urine samples [11]. Our previous study [12] showed that DEK protein expression was closely related with the proliferation of both ovarian and breast cancers, and that its over expression was significantly correlated with the increased Ki-67 proliferation index in uterine cervical cancers. These studies suggest that DEK activities may be essential for cancer progression. Therefore, DEK depletion has been suggested as a novel therapeutic method for cancer-targeted therapy.

However, to date, the expression status of DEK in colorectal cancer and its relationship with clinicopathological features/prognosis is unknown [13-15]. To determine whether DEK is important in the tumorigenesis of colorectal cancers and investigate its prognostic value, 109 cases of colorectal cancer and 52 colorectal adenoma tissues were selected for the analysis of DEK by immunohistochemical staining. Additionally, the prognostic significance of carcinoembryonic antigen (CEA), a well-established prognostic factor for colorectal cancer, was also analyzed to verify the reliability of this cohort of colorectal cancer patients. Our data uncovered that DEK is frequently upregulated in colorectal cancers when compared with either the normal tissues counterparts or colorectal adenomas. These findings suggest that DEK may be an independent predictor for poor prognosis in patients with colorectal cancer.

## Methods

### Ethics statement

This study complied with the Helsinki Declaration and was approved by the Human Ethics the Research Ethics committees of the Dandong Center Hospital of China. Through the surgery consent form, patients were informed that the resected specimens were kept by our hospital and might be used for scientific research, and that their privacy would be maintained. Follow-up survival data were collected retrospectively through medical-record analyses.

### Tissue specimens and follow-up observation

The routinely processed and diagnosed colorectal cancer tissues (109 cases) with strict follow-up were randomly selected from the patients who underwent surgery between 2004 and 2007 in the Dandong Center Hospital of China. Pathological parameters, including age, gender, grade, nodal metastasis, clinical stage and survival data, were carefully reviewed in all cases. The patients’ ages ranged from 34 to 76 years with a mean age of 48.6 yrs. The male to female ratio was 87:22. The tumor location was categorized as colonic and ileocecal in 57 cases, and rectal in 52 cases. The hematoxylin and eosin-stained slides of the different biopsies were reviewed by two experienced pathologists and one appropriate paraffin block was selected for this study. Staging was performed according to the TNM and FIGO classification of carcinoma of the colon and rectum. From these 109 tumor tissues, 59 were FIGO stage I-IIA, which is considered early stage. Fifty samples were stage IIB-IIIC, an advanced stage according to the Union for International Cancer Control 7th Edition criteria and the World Health Organization classification (Pathology & Genetics Tumors of the digestive system) [16]. Of the 109 cases, 49 were well-differentiated and 60 were poorly differentiated cancers. Adjacent normal colon mucosa tissues from the cancer resection margin and 52 colorectal adenoma tissues were also included in this study. Before surgery, no patients had received chemotherapy or had distant metastases, and all patients had serum CEA detection (0-5 μg/ml as normal). The 109 cancer patients were followed-up for survival. By March 2012, 39 patients had died while 70 patients remained alive. The median survival time was 56 months.

### Immunohistochemistry for DEK in paraffin-embedded tissues

Immunohistochemical analysis was performed using the DAKO LSAB kit (DAKO A/S, Glostrup, Denmark). Briefly, to eliminate endogenous peroxidase activity, 4 μm thick tissue sections were deparaffinized, rehydrated and incubated with 3% H_2_O_2_ in methanol for 15 min at room temperature (RT). The antigen was retrieved at 95°C for 20 min by placing the slides in 0.01 M sodium citrate buffer (pH 6.0). The slides were then incubated with DEK antibody (1:50, BD Biosciences Pharmingen, CA, USA) at 4°C overnight. After incubation with biotinylated secondary antibody at RT for 30 min, the slides were incubated with streptavidin-peroxidase complex at RT for 30 min. Immunostaining was developed by using 3,3′-diaminobenzidine, and Mayer’s hematoxylin was used for counterstaining. We used tonsil sections as the positive controls and Mouse IgG as an isotope controls. In addition, the positive tissue sections were processed with omitting of the primary antibody (mouse anti-DEK) as negative controls.

### Evaluation of immunohistochemical staining

All specimens were examined by two pathologists (Lin Z & Liu S) who did not possess knowledge of the clinical data. In case of discrepancies, a final score was established by reassessment on a double-headed microscope. Briefly, the immunostaining for DEK was semi-quantitatively scored as ‘-’ (negative, no or less than 5% positive cells), ‘+’ (5–25% positive cells), ‘++’ (26–50% positive cells) and ‘+++’ (more than 50% positive cells). Only the nuclear expression pattern was considered as positive staining. The strongly positive descriptor (DEK over expression) was assigned to ‘++’ and ‘+++’ scored cells. For survival analysis, DEK expression level was denoted as high expression (‘++’ and ‘+++’) and low expression (‘-’ and ‘+’).

### Statistical analysis

Statistical analyses were performed using the SPSS 17.0. Correlation between DEK expression and clinicopathological characteristics were evaluated by Chi-square test and Fisher’s exact tests. The survival rates after tumor removal were calculated by the Kaplan-Meier method, and differences in survival curves were analyzed by the Log-rank tests. Multivariate survival analysis was performed on all the significant characteristics measured by univariate survival analysis (gender, age, tumor size, differenciation, lymph node metastasis, serosal invasion, tumor stage, CEA level, and DEK expression) through the Cox proportional hazard regression model. *P<*0.05 was considered statistically significant.

The variables such as CEA level, DEK expression, stages, and differentiations were grouped two as normal vs increased level of CEA, high expression vs low expression of DEK, early stage (I-IIA) vs late stage (IIB-IIIC), and well vs poorly and moderately differentiated, respectively.

## Results

### DEK protein is over expressed in colorectal cancer

**Figure 1:**
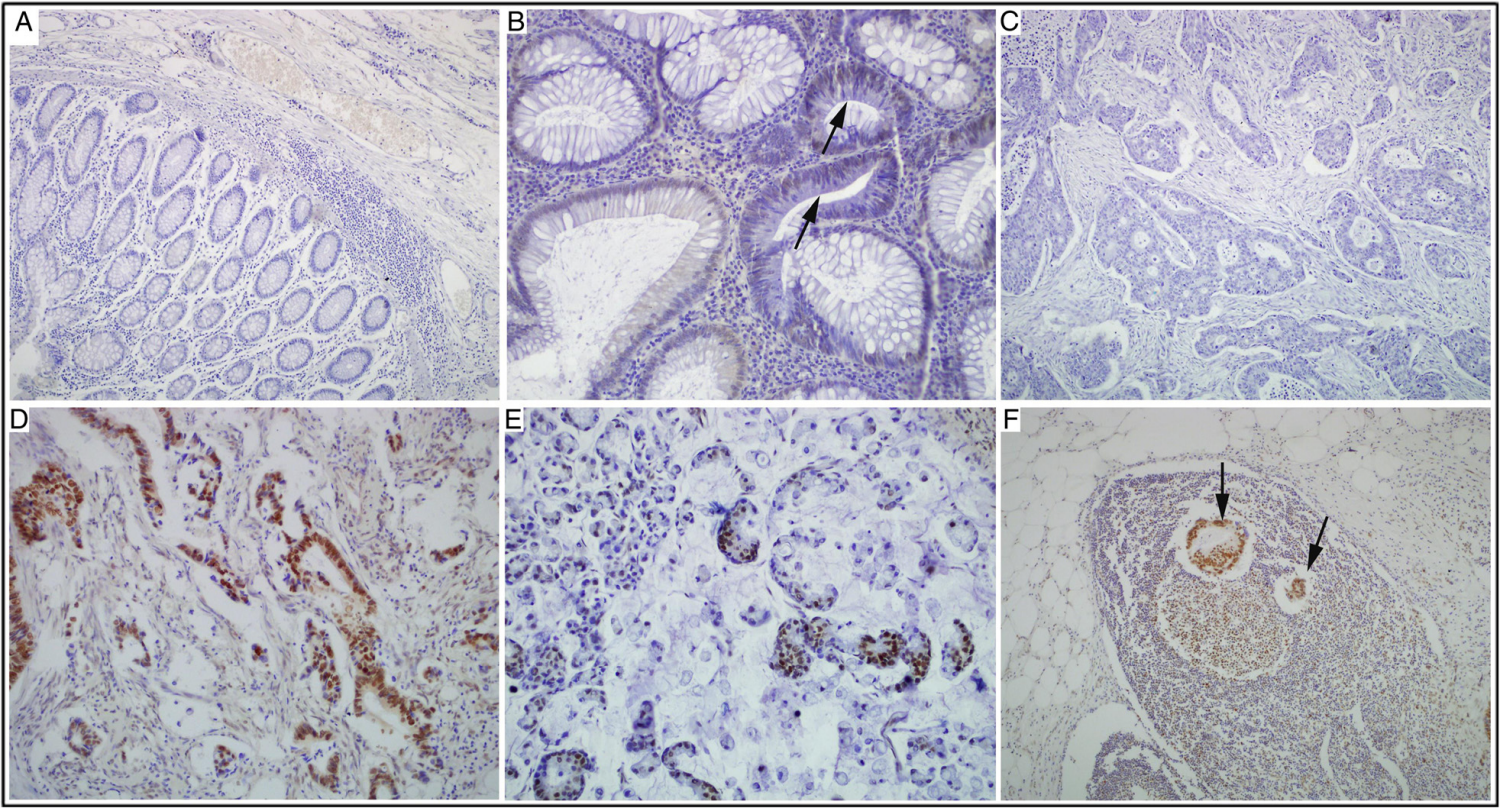
**Immunohistochemical staining of DEK protein in colorectal cancer, adenoma, and normal mucosa. (A)** DEK is absolutely negative for DEK protein in normal colorectal mucosa (Original magnification, ×100). **(B)** DEK is positive in the dysplastic cells of colorectal adenomas (Original magnification, ×200). **(C)** DEK is negative in colorectal cancer without lymph node metastasis (Original magnification, ×200). **(D)** DEK is strongly positive in the cancer cells of colorectal cancer with lymph node metastasis (Original magnification, ×200). **(E)** DEK is positive in the signet ring cells of colorectal cancers. **(F)** DEK is strongly positive in the metastatic cancer cells (arrows) in lymph node (Original magnification, ×100).

DEK protein expression showed a nuclear immunohistochemical staining pattern in colorectal cancers (Figure 1). The positive rate of DEK protein expression was significantly higher in colorectal cancer tissues (95.41%, 104/109) than in either normal adjacent mucosa (33.03%, 36/109) or colorectal adenomas (32.69%, 17/52). Similarly, the strongly positive rate of DEK protein was 48.62% (53/109) in colorectal cancers, which was significantly higher than that in either adjacent normal colon mucosa (9.17%, 10/109) or colorectal adenomas (13.46%, 7/52) (P<0.01, respectively) (Table 1).

**Table 1:** DEK protein expression in colorectal adenocarcinoma

**Diagnosis**	**No. of cases**	**DEK expression**				**Positive rate (%)**	**Strongly positive rate (%)**
		**-**	**+**	**++**	**+++**		
Normal	109	73	26	10	0	33.03%	9.17%
Adenoma	52	35	10	7	0	32.69%	13.46%
Cancer	109	5	51	23	30	95.41%**	48.62%**

**P<0.01, compared with peripheral normal mucosa and adenomas of colon.

Adenoma: colorectal adenoma; Cancer: colorectal cancer;

Positive rate: percentage of positive cases with ‘+’, ‘++’, and ‘+++’ staining score;

Strongly positive rate: percentage of positive cases with ‘++’ and ‘+++’ staining score.

### Clinicopathological and prognostic significance of DEK over expression

**Figure 2:**
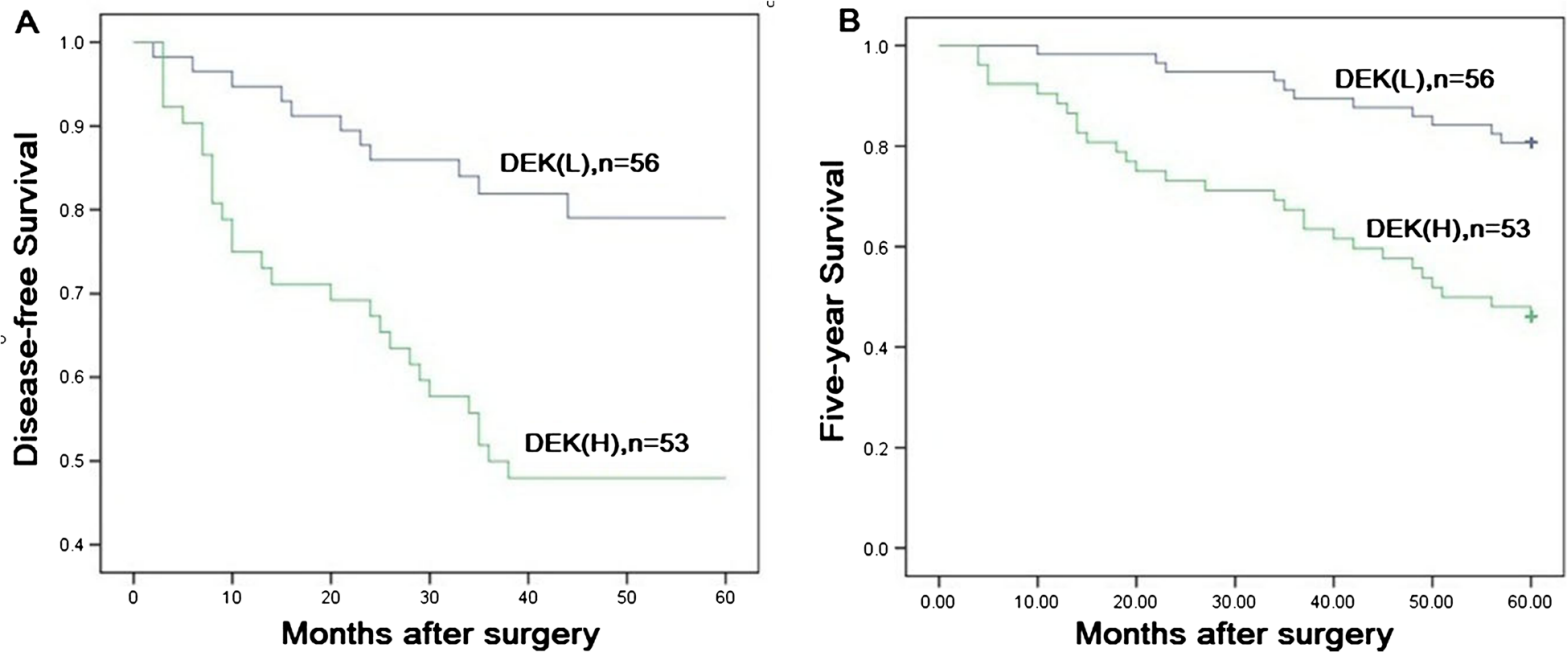
**Kaplan-Meier analyses of disease-free and 5-year survival rates in 109 colorectal cancer patients in relation to DEK protein over expression.** Patients with colorectal cancer with high DEK expression had lower disease-free (**A**, P<0.0001) and 5-year (**B**, P<0.0001) survival rates than those with without high DEK expression as determined using the Kaplan-Meier method. (H, high; L, low).

To evaluate the relationship between DEK protein and colorectal cancer progression, we analyzed the correlation between DEK protein over expression and clinicopathological features of colorectal cancers. The strongly positive rate of DEK protein was significantly higher in colorectal cancers with >5 cm tumor size than in cases with ≤5 cm tumor size (P=0.029). Similarly, we found that the strongly positive rate of DEK protein was significantly higher in colorectal cancers with lymph node metastasis (63.27%, 31/49) than in cases without metastasis (36.67%, 22/60) (P=0.006). It was also higher in poorly and moderately differentiated colorectal cancers (60.00%, 36/60) than in well-differentiated cases (34.69%, 17/49) (P=0.009). For the TNM and FIGO clinical stages, the strongly positive rate of DEK protein was 62.00% (31/50) in the advanced stage (IIB–IIIC) colorectal cancers, but only 37.29% (22/59) in early stage cases (I–IIA) (P=0.010). Meanwhile, the strongly positive rate of DEK protein was higher in cancer cases with serosal invasion (50.00%, 26/52) than in those with no serosal invasion (P=0.031). However, the over expression of DEK protein was not related with gender, age, tumor location or CEA levels of patients with colorectal cancer (Table 2). Moreover, patients with colorectal cancer with high DEK expression had lower disease-free and 5-year survival rates than those without high DEK expression as determined using the Kaplan-Meier method (P<0.0001) (Figure 2A-B).

**Table 2:** Chi-square test and Fisher’s exact test of relationship between DEK over expression and the clinicopathological features in colorectal cancers

**Characteristic**	**No. of cases**	**Strongly positive cases (%)**	**OR (95% CI)**	***P *value**
**Gender**			2.461 (0.924-6.556)	0.067
Male	87	45 (51.72%)		
Female	22	8 (36.36%)		
**Age (years old)**			1.298 (0.611-2.756)	0.497
≥49	53	24 (45.28%)		
<49	56	29 (51.79%)		
**Tumor size (cm)**			2.353 (1.086-5.101)	0.029
≤5	61	24 (39.34%)		
>5	48	29 (60.42%)		
**Location**			0.900 (0.424-1.910)	0.784
Colonic & ileocecal	57	27 (47.37%)		
Rectal	52	26 (50.00%)		
**Differentiation**			2.824 (1.291-6.177)	0.009
Well diff.	49	17 (34.69%)		
Poorly & mod. diff.	60	36 (60.00%)		
**Lymph node metastasis**			2.975 (1.360-6.509)	0.006
-	60	22 (36.67%)		
+	49	31 (63.27%)		
**Serosal invasion**			2.353 (1.072-5.163)	0.031
-	57	17 (29.82%)		
+	52	26 (50.00%)		
**Stage**			2.744 (1.261-5.971)	0.010
I-IIA	59	22 (37.29%)		
IIB-IIIC	50	31 (62.00%)		
**CEA**			0.610 (0.273-1.362)	0.228
Normal	37	15 (40.54%)		
Increased	72	38 (52.78%)		

**Figure 3:**
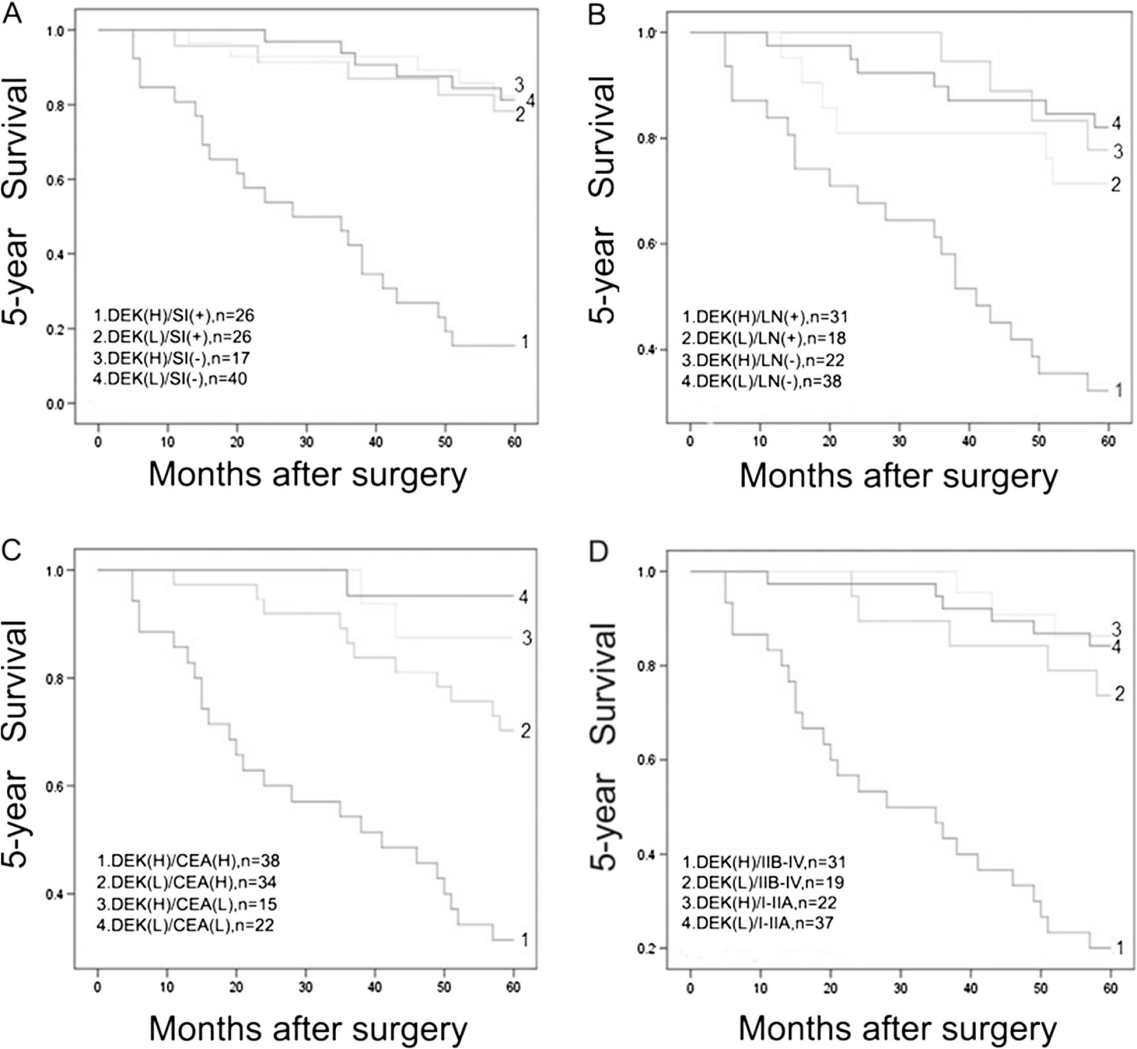
**Kaplan-Meier analysis of 5-year survival rates in 109 patients with or without DEK highly expressed colorectal cancer in relation to serosal invasion (SI), lymph node (LN) metastasis, CEA level, and tumor stage. (A)** Colorectal cancer with serosal invasion concomitant with DEK expression had a significantly lower 5-year survival rate than that without DEK expression (P<0.0001). **(B)** Colorectal cancer with lymph node metastasis and high DEK expression, had a significantly lower 5-year survival rate than colorectal cancer with lymph node metastasis in the absence of DEK expression (P=0.001). **(C)** Colorectal cancer patients with high CEA levels concomitant with high DEK expression had lower 5-year survival rates than those without DEK expression (P<0.0001). **(D)** Late-stage colorectal cancers concomitant with high DEK expression had the lowest 5-year survival rate, which was significantly lower than those without high DEK expression (P=0.004). (H, high; L, low).

To further substantiate the importance of high DEK expression in colorectal cancer progression, we compared its effect on prognosis by analyzing the correlations between DEK expression and factors associated with aggressiveness of colorectal cancer. Serosal invasion, lymph node metastasis, CEA level and tumor stage were all associated with lower 5-year survival rates (P<0.001). By combination analysis, (Figure 3A), we found that colorectal cancer with serosal invasion concomitant with DEK expression had a significantly lower 5-year survival rate than that without DEK expression (P<0.0001). Similarly, colorectal cancer with lymph node metastasis and high DEK expression, had a significantly lower 5-year survival rate than colorectal cancer with lymph node metastasis in the absence of DEK expression (Figure 3B, P=0.001). In addition, colorectal cancer patients with high CEA levels concomitant with high DEK expression had lower 5-year survival rates than those without DEK expression (Figure 3C, P<0.0001). Most importantly, late-stage colorectal cancers concomitant with high DEK expression had the lowest 5-year survival rate, which was significantly lower than those without high DEK expression (Figure 3D, P=0.004).

### DEK over expression is an independent prognostic factor in colorectal cancers by Cox proportional hazard regression model

Using univariate analysis, we found that colorectal cancer patients with DEK over expression had significantly lower 5-year survival rates than those without DEK-overexpressing tumors. Additionally, serosal invasion, tumor stage, and CEA level were also associated with 5-year survival rates when DEK was expressed (Table 3). These data suggest that DEK could also be a valuable prognostic factor in colorectal cancer. Therefore, multivariate analysis was performed using the Cox proportional hazards model for all of the significant variables examined in the univariate analysis. We found that serosal invasion (HR: 1.708, 95% CI: 1.414–2.555, P=0.009) and late stage (HR: 1.663, 95% CI: 1.081–2.558, P=0.021) proved to be independent prognostic factors for survival in colorectal cancer (Table 4). This result validates the clinical application that elevated CEA level and serosal invasion predict poor survival of patients with colorectal cancer. Importantly, DEK over expression emerged as a significant independent prognostic factor in colorectal cancer (HR: 1.805, 95% CI: 1.208–2.699, P=0.004) (Table 4).

**Table 3:** Univariate survival analyses (Cox regression model) of various factors in patients with colorectal cancer

**Factors**	**B**	**SE**	**Wald**	**HR**	**95% CI**		**P value**
					**Lower**	**Upper**	
**Gender**	0.235	0.239	0.971	1.266	0.445	0.946	0.324
**Age**	0.266	0.192	1.908	1.304	0.895	1.901	0.167
**Tumor size**	0.059	0.193	0.093	1.061	0.727	1.548	0.760
**Location**	0.024	0.192	0.016	1.025	0.704	1.492	0.899
**Differentiation**	0.265	0.193	0.736	1.180	0.809	1.722	0.391
**Lymph node metastasis**	0.374	0.193	3.756	1.454	0.996	2.123	0.053
**Serosal invasion**	0.437	0.192	5.155	1.547	1.062	2.256	0.023*
**Tumor stage**	0.741	0.194	14.573	2.098	1.434	3.069	<0.0001*
**CEA**	0.475	0.203	5.468	1.607	1.080	2.392	0.019*
**DEK**	0.432	0.192	5.050	1.540	1.057	2.246	0.025*

*B* Coefficient.

*SE* standard error.

*Wald* Wald statistic.

*HR* hazard ratio.

*CI* confidence interval.

*Significant different.

**Table 4:** Multivariant survival analyses (Cox regression model) of various factors in patients with colorectal cancer

**Factors**	**B**	**SE**	**Wald**	**HR**	**95% CI**		**P value**
					**Lower**	**Upper**	
**Serosal invasion**	0.535	0.206	6.778	1.708	1.141	2.555	0.009*
**Tumor stage**	0.509	0.220	5.366	1.663	1.081	2.558	0.021*
**CEA**	0.347	0.228	2.305	1.415	0.904	2.214	0.129
**DEK**	0.591	0.205	8.290	1.805	1.208	2.699	0.004*

*B* Coefficient.

*SE* standard error.

*Wald* Wald statistic.

*HR* hazard ratio.

*CI* confidence interval.

*Significant different.

## Discussion

Colorectal cancer is the most common malignancy of the gastrointestinal tract [17]. It causes 655,000 deaths worldwide every year [18]. As a high-risk and highly metastatic cancer, the identification of reliable criteria for predicting recurrence and for identifying colorectal tumors is of great interest not only for understanding the molecular and cellular processes involved, but also for uncovering possible new therapeutic molecular targets.

DEK was discovered by the identification of translocation t(6;9) (p23;q34) in a subset of patients with AML. In fact, this translocation has been considered for use in AML patient stratification. Chromosomal alterations at the DEK locus are now known not to be a universal feature of malignancy, even in AML. However, the increasing list of tumor types, including AML [19,20], glioblastoma [21], hepatocellular carcinoma [22], melanoma [23], ovarian cancer [12], cervical cancer [24] and others [25-27], showing high DEK protein expression raises the exciting possibility of using DEK as a tumor marker [6]. Kappes et al. investigated the localization of DEK throughout the cell cycle and found it was always on chromatin and as a component of mitotic chromosomes [28]. Khodadoust et al. reported that DEK expression levels can distinguish benign nevi from malignant melanomas, indicating that this protein may prove to be highly useful for differentiating diagnosis [29]. This is a prime example of a clinically relevant setting in which this protein may prove to be highly useful. Trisha et al. used littermate DEK knockout, heterozygous and wild type mice for their experiments, and found that there was a significant delay in the formation of papillomas in DEK knockout mice compared with wild type and heterozygous mice. Our previous data also showed that DEK protein was strongly positive in breast cancers and DCIS (ductal carcinoma in situ), but negative in normal breast glands, demonstrating that DEK protein expression levels might be used as a biomarker for early diagnosis of breast cancers [30].

Babaei-Jadidi R et al. reported that accumulation of DEK and loss of epithelial TPM may contribute to the oncogenicity of FBXW7 mutation in both human colorectal cancer and in the Apc^Min/+^/Fbxw7^ΔG^ mouse intestine, which is indicative of possible roles for DEK and TPM in colorectal tumorigenesis. And DEK expressions in epithelial cells are correlated with FBXW7 mutations in human colorectal cancer [31]. However, DEK protooncogene function and the regulation of its expression levels are largely unclear. The overall goal of this study was to determine whether the over expression of DEK oncoprotein might serve as a biomarker for the prognostic evaluation of colorectal cancers. This is the first study, to our knowledge, to correlate DEK levels in colorectal cancers with histological prognostic factors to understand the role of DEK up regulation in colorectal cancer progression. Here we performed immunohistochemical staining of DEK protein and survival data analysis using 52 of colon adenomas and 109 of colorectal adenocarcinomas and their adjacent normal tissue counterparts. We found that the positive and strongly positive rates of DEK oncoprotein were significantly higher in colorectal cancers than those for either adjacent normal tissues or adenomas. These findings indicate that DEK potentially plays important roles in the progression of colorectal cancer (Table 1).

Moreover, as DEK may be present at higher levels in immature cells than in differentiated counterparts, it could also aid in gauging the differentiation potential of tumor cells. Kavanaugh et al. reported that DEK over expression promotes the transformation of human keratinocytes, and that DEK knockout mice are partially resistant to chemically induced papilloma formation [32]. Shibata et al. also showed that DEK over expression, partly through an increase in its gene dose, mediates the activity of global transcriptional regulators and is associated with tumor initiation activity and poor prognosis in high-grade neuroendocrine carcinoma [33]. Here we demonstrate that DEK over expression correlated with large tumor size, low differentiation, serosal invasion, lymph node metastasis, and late-stage in colorectal carcinomas. However, DEK expression level was not correlated with gender, age, tumor location or CEA level in patients with colorectal cancers (Table 2). These results indicate that DEK might be a new attractive molecular target for therapy.

Despite the strong association between DEK expression and cancer, reports of DEK expression-based outcome in tumor patients are limited. Using SAGE (Serial Analysis of Gene Expression) and real-time polymerase chain reaction (PCR), Abba et al. found that DEK and DCTN3 are significantly over expressed in breast carcinomas with lymph node metastasis or poor prognosis [34]. Privette Vinnedge et al. demonstrated that DEK expression is associated with positive hormone receptor status in primary breast cancers and is up-regulated in vitro following exposure to the hormones estrogen, progesterone, and androgen. Moreover, chromatin immunoprecipitation experiments identified DEK as a novel estrogen receptor-α target gene whose expression promotes estrogen-induced proliferation. These data suggest that DEK promotes the pathogenesis of ER+ breast cancer and that the targeted inhibition of DEK may enhance the efficacy of conventional hormone therapies [2]. Similarly, our previous study reported that the strongly positive rate of DEK protein was significantly higher in breast cancers with <3 years disease-free survival than in cases with ≥3 years disease-free survival, suggesting that the detection of >25% DEK expression levels could play a role as a marker of poor prognosis in breast cancer [30]. Here we have demonstrated that high DEK expression is associated with serosal invasion, lymph node metastasis, tumor size and differentiation, which are crucial histological features associated with poor prognosis in colorectal cancer. We demonstrated that colorectal cancers exhibiting serosal invasion, lymph node-positivity, and elevated CEA had lower 5-year survival rates. Importantly, DEK over expression concomitant with any of these features correlated with significantly lower 5-year survival rates than those without DEK expression. Of particular interest, high DEK expression was found to be an independent hazard factor in colorectal cancer. These findings raise the possibility that DEK not only facilitates serosal invasion, lymph node metastasis, and CEA elevation but also aggressive cancer behavior, resulting in poor prognosis for patients. Importantly, we demonstrated that colorectal cancer with high DEK expression correlated with late-stage tumors. Tumor stage is an independent prognostic factor in other studies and as well as in our study [16,35] (Figure 3 and Tables 3 and 4).

## Conclusion

In conclusion, we have identified DEK as a potential biomarker for evaluation of tumor progression and prognosis of colorectal cancers. DEK expression was more commonly seen in cases presenting with poor prognostic factors of colorectal cancer, leading to lymph node metastasis, late-stage, serosal invasion and reduced survival time. Further studies are warranted to more firmly establish this supposition.

## Competing interests

The authors declare that they have no competing interests.

## Authors’ contributions

LL, PJ, and GW participated in study conception, design, case selection and immunohistochemical staining. MY, JG and LL carried out the data collection. LJ, PY and LZ performed the scoring of immunohistochemical staining. LL and LZ performed data analysis and writing of the manuscript. All the authors read and approved the final manuscript.

## Pre-publication history

The pre-publication history for this paper can be accessed here:

http://www.biomedcentral.com/1471-2407/13/366/prepub
